# Acute pulmonary Edema following Myometrial pituitrin injection during laparoscopic myomectomy

**DOI:** 10.1093/omcr/omag038

**Published:** 2026-04-14

**Authors:** Zhao Wang, Shan Song, Jing Zhang

**Affiliations:** Department of Anesthesiology, The Affiliated Yantai Yuhuangding Hospital of Qingdao University, No. 20 Yuhuangding East Road, Zhifu District, Yantai, Shandong 264000, China; Department of Anesthesiology, The Affiliated Yantai Yuhuangding Hospital of Qingdao University, No. 20 Yuhuangding East Road, Zhifu District, Yantai, Shandong 264000, China; Department of Anesthesiology, The Affiliated Yantai Yuhuangding Hospital of Qingdao University, No. 20 Yuhuangding East Road, Zhifu District, Yantai, Shandong 264000, China

**Keywords:** pituitary hormones, posterior, pulmonary Edema, heart failure, laparoscopy, myomectomy, intraoperative complications

## Introduction

Pituitrin, an extract of posterior pituitary hormones, is routinely used in gynecological surgery to minimize bleeding through its vasoconstrictive components [1]. However, systemic absorption of vasopressin may induce serious cardiopulmonary complications, including acute pulmonary edema [2]. While previous case reports have described cardiovascular events associated with pituitrin, comprehensive documentation with real-time echocardiographic evidence and detailed mechanistic analysis remains limited [3]. This report presents a well-documented case of pituitrin-induced acute pulmonary edema, providing information on intraoperative echocardiographic imaging, serial biomarker trends, and a detailed pathophysiological explanation to enhance clinical recognition and inform safer clinical practices.

## Case report

A 53-year-old female patient (72 kg, 165 cm, ASA of Anesthesiologists Physical Status II) with multiple uterine fibroids underwent elective laparoscopic myomectomy at our medical facility. Preoperative assessments, including electrocardiography and transthoracic echocardiography, confirmed normal cardiac function (ejection fraction > 60%) and the absence of underlying heart disease. The patient had no cardiac risk factors, such as hypertension, diabetes mellitus, or a smoking history.

The preoperative vital signs were as follows: blood pressure, 126/81 mmHg; heart rate, 69 beats per minute (bpm); oxygen saturation, 100%; temperature, 36.7°C; and respiratory rate, 20 breaths/min. General anesthesia was induced intravenously using midazolam (0.03 mg/kg), etomidate (0.3 mg/kg), sufentanil (0.5 μg/kg), and cis-atracurium (0.15 mg/kg). Following tracheal intubation, anesthesia was maintained with sevoflurane (1.5–2.5 vol%) and a continuous infusion of remifentanil (0.05–0.2 μg/kg/min). According to the institutional protocol, pituitrin was diluted in 0.9% normal saline to a concentration of 0.15 U/ml. A total volume of 40 mL (total dose of 6 U) was injected into the myometrium at multiple sites. The temporal sequence of events was as follows: Within two minutes of the injection, the patient demonstrated significant bradycardia, with the heart rate decreasing from 70 to 38 beats per minute, and hypertension, as indicated by an increase in systolic blood pressure to 180 mmHg. Atropine (0.5 mg) and propofol (100 mg) administration resulted in temporary stabilization. However, a decline in hemodynamic parameters was observed 10 min after injection. At 15 min post-injection, the oxygen saturation decreased to 90%, accompanied by elevated airway pressure. Pink, frothy sputum emerged from the endotracheal tube and was associated with bilateral rales on chest auscultation.

**Figure 1:**
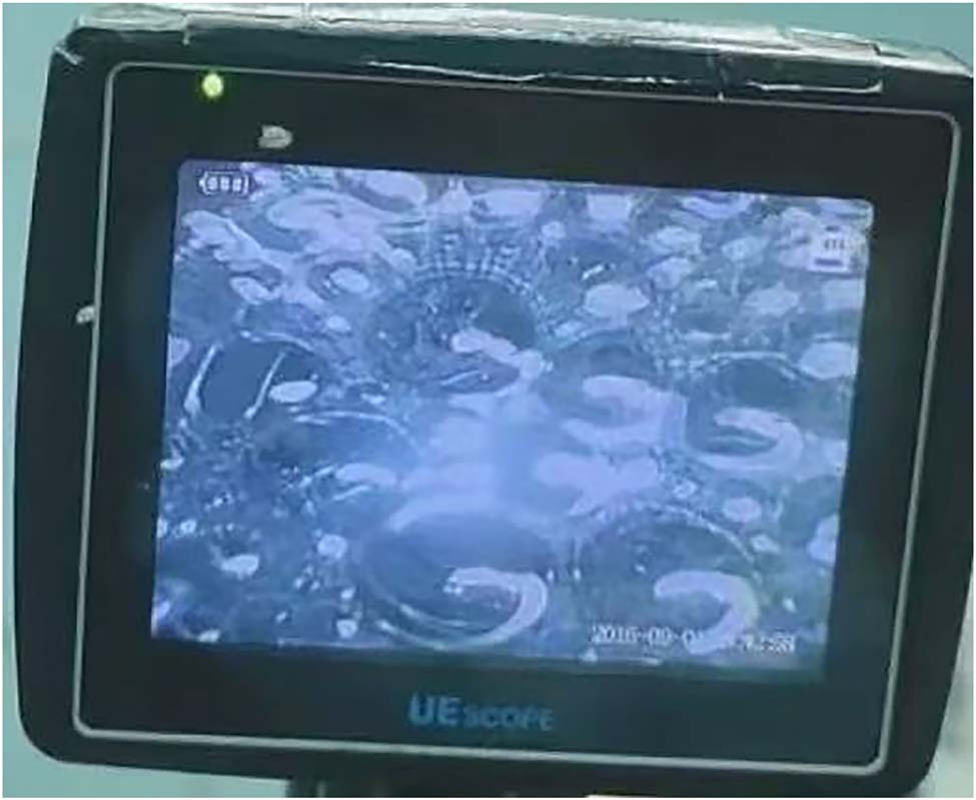
Fiberoptic bronchoscopy reveals abundant frothy sputum in the tracheal lumen.

**Figure 2:**
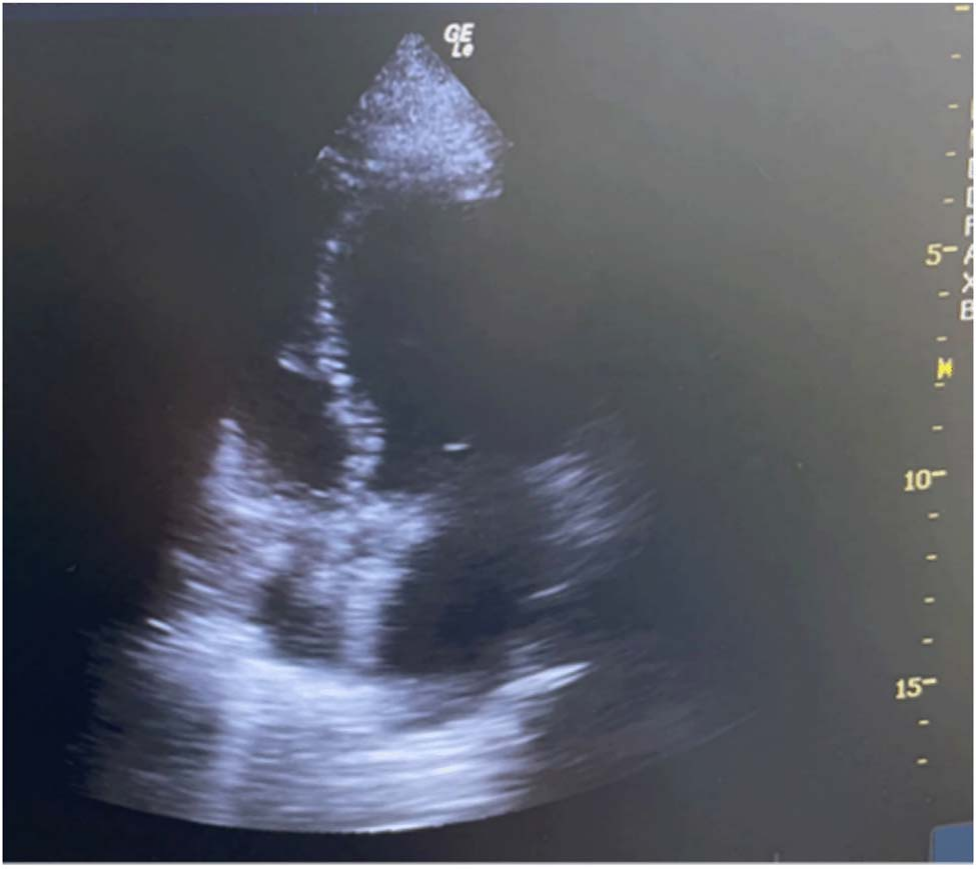
Bedside echocardiography revealed left ventricular dilation, diffuse hypokinesia, and a reduced ejection fraction of 30%.

**Figure 3:**
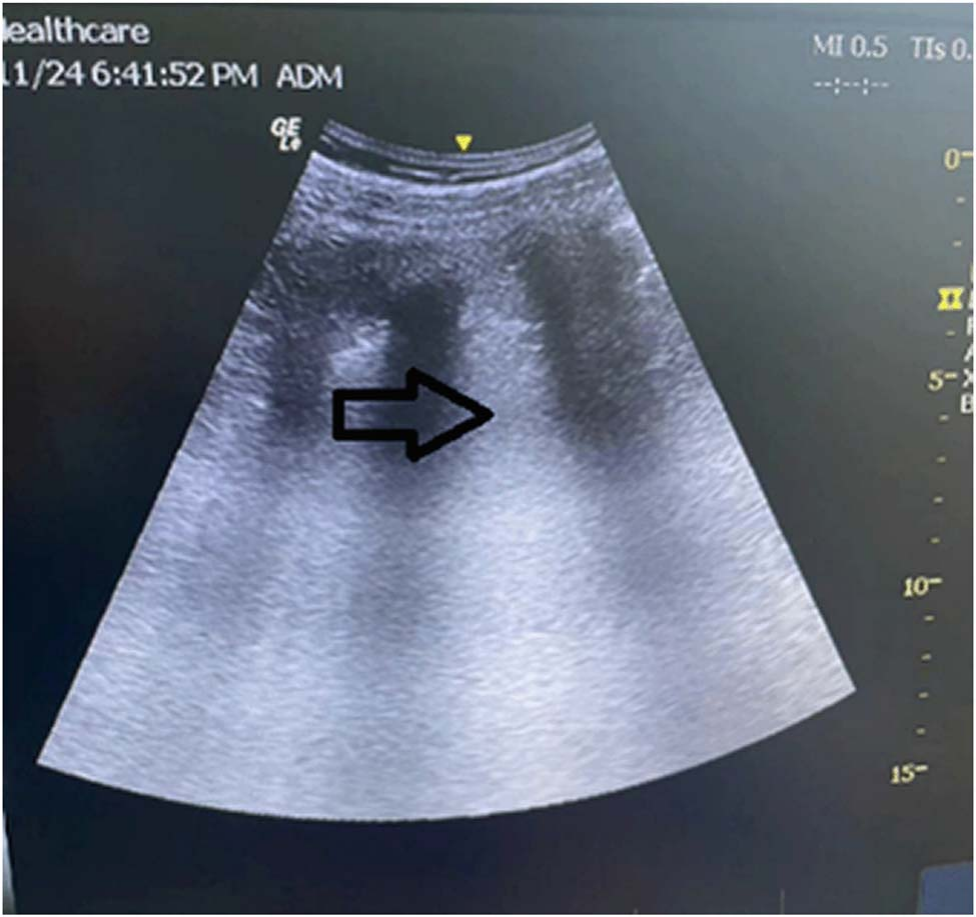
Lung ultrasonography revealed diffuse B-lines consistent with pulmonary edema.

Fiberoptic bronchoscopy reveals abundant frothy sputum in the tracheal lumen (Fig. 1). The patient was diagnosed with acute pulmonary edema and was treated with intravenous furosemide (20 mg), restricted fluid intake (limiting total crystalloids to 700 mL), invasive arterial pressure monitoring, and vasopressor support through dopamine and epinephrine infusions. Bedside echocardiography revealed left ventricular dilation, diffuse hypokinesia, and a reduced ejection fraction of 30% (Fig. 2). Lung ultrasonography revealed diffuse B-lines consistent with pulmonary edema (Fig. 3). Other potential diagnoses were systematically considered and excluded. Fluid overload was considered unlikely given the minimal pre-event fluid administration (500 ml). Anaphylaxis was ruled out because of the absence of rashes or bronchospasms. Finally, pulmonary embolism was excluded based on echocardiographic findings of isolated left ventricular dysfunction without any signs of a right ventricular strain.

The patient was transferred to the intensive care unit (ICU) and extubated after three hours, following satisfactory weaning parameters. Serial cardiac monitoring revealed a troponin-I peak of 1835.5 ng/ml (normal < 0.04 ng/ml) at 12 h, which declined to 426 ng/ml by day three. Additionally, B-type natriuretic peptide (BNP) peaked at 427.9 pg/ml (normal < 100 pg/ml) at 12 h and decreased to 237.18 pg/mL by day three. Cardiac function was normalized at discharge on day five, as confirmed by a follow-up one month later.

## Discussion

This case provides compelling evidence of pituitrin-induced acute pulmonary edema in a patient with normal preoperative cardiac function. The rapid symptom onset (2–15 minutes post-injection) strongly supports a direct drug effect.

Pathophysiological Mechanism: We propose a tripartite mechanism for this phenomenon. First, systemic vasopressin absorption induces coronary vasospasm, leading to myocardial ischemia, as evidenced by elevated troponin levels and ventricular hypokinesia [4]. Second, potent vasoconstriction increases left ventricular afterload (SBP 180 mmHg), precipitating systolic dysfunction [5]. Third, elevated left ventricular pressure is transmitted retrograde, leading to hydrostatic pulmonary edema [6]. Pneumoperitoneum and Trendelenburg positioning may exacerbate these effects [7].

## Dosing and risk mitigation

Pituitrin, a posterior pituitary extract containing vasopressin and oxytocin, was used at a total dose of 6 U (0.15 U/ml), a concentration supported by the surgical literature [8, 9]. Although effective for hemostasis, the systemic absorption of its vasopressin component can induce coronary and systemic vasoconstriction, increasing afterload and precipitating acute heart failure, as seen in our patient. In contrast, Pitocin (synthetic oxytocin) promotes uterine contractions with minimal cardiovascular risk. Thus, when uterine hemostasis is required, particularly in patients with cardiac concerns, Pitocin is a safer alternative to Pituitrin.

### Clinical implications

This case adds to previous reports by providing comprehensive echocardiographic and biomarker documentation of evolving cardiac injury [2, 3]. This highlights that this complication can occur even in the absence of pre-existing heart disease. Clinicians should consider alternatives such as misoprostol and emphasize interdisciplinary communication [10].

Comparison with Literature: Our findings are consistent with recent reports of pituitrin-related complications but provide more detailed mechanistic insights through comprehensive intraoperative monitoring.

### Limitations

As this was a single case report, the findings cannot be generalized to a larger population. Although evidence strongly supports pituitrin as the cause, idiosyncratic reactions cannot be completely excluded. Coronary angiography was not performed in this patient. Future studies should quantify this risk to establish definitive safety guidelines.

### Conclusion

This case demonstrates that myometrial pituitrin injection can cause life-threatening pulmonary edema, even in patients with normal cardiac function. Strict dose limitations, vigilant monitoring, and awareness of potential complications are essential to ensure patient safety are essential.

The authors express their sincere gratitude to the multidisciplinary team involved in this patient's critical care management, including the anesthesiology, gynecologic surgery, and intensive care unit staff at our institution. We thank Dr. JiangLin Cong and Dr. Xiuli Wang from the gynecologic surgery team for their decisive intervention and skilled performance of the urgent surgery. We thank Dr. Minghui Sun, Department of Ultrasound, for expert echocardiographic interpretation, and Dr. Jia Liu, Department of Intensive Care Unit, for postoperative monitoring and treatment. Special acknowledgment was given to the nursing team for their vigilant postoperative monitoring of patients. The patient provided written consent for the publication of this report. No external funding or editorial assistance was received for this study.

## References

[1] Chudnoff S, Glazer S, Levie M. Review of vasopressin use in Gynecologic surgery. J Minim Invasive Gynecol 2012;19:422–33. 10.1016/j.jmig.2012.03.022 22748950

[2] Shinn HK, Song JH, Han JW. et al. Cardiac arrest caused by Intramyometrial infiltration of vasopressin during dilation and curettage under general Anesthesia - a case report. Korean J Anesthesiol 2007;53:664. 10.4097/kjae.2007.53.5.664

[3] Kim JW, Kim G, Kim TW. et al. Hemodynamic changes following accidental infiltration of high-dose vasopressin. J Int Med Res 2020;48:1–7. 10.1177/0300060520959494 PMC7536500 32993389

[4] Li JR, Liao X, Lv FQ. et al. Cardiac arrest caused by pituitrin administration during laparoscopic myomectomy: a case report. BMC Womens Health 2023;23:111. 10.1186/s12905-023-02255-w 36934254 PMC10024353

[5] Pelletier JS, Dicken B, Bigam D. et al. Cardiac effects of vasopressin. J Cardiovasc Pharmacol 2014;64:100–7. 10.1097/fjc.0000000000000092 24621650

[6] Marteles MS, Urrutia A. Formas de presentación de la insuficiencia cardíaca aguda: edema pulmonar Agudo y shock cardiogénico. Med Clin (Barc) 2014;142:14–9. 10.1016/s0025-7753(14)70077-6 24930078

[7] Udeh CI, Diaz-Gomez JL, Satyapriya D. et al. Recent advances in perioperative Anesthetic management: an update on the role of vasopressin and its effects on patient outcomes. Curr Pharm Des 2012;18:6308–13. 10.2174/138161212803832326 22762470

[8] Suh MK, Seong KW, Jung SH. et al. Effect of pneumoperitoneum and Trendelenburg position on respiratory mechanics during laparoscopic surgery. Korean J Anesthesiol 2010;59:329–34. 10.4097/kjae.2010.59.5.329 21179295 PMC2998653

[9] Guo F, Jiao C, Xu K. et al. Optimal dose of pituitrin in laparoscopic uterine myomectomy: a double-blinded, randomized controlled trial. J Minim Invasive Gynecol 2021;28:2073–9. 10.1016/j.jmig.2021.06.008 34147692

[10] Dabash R, Blum J, Raghavan S. et al. Misoprostol for the management of postpartum bleeding: a new approach. Int J Gynaecol Obstet 2012;119:210–2. 10.1016/j.ijgo.2012.08.005 22980431

